# Inhibition of Poly-ADP-Ribosylation Fails to Increase Axonal Regeneration or Improve Functional Recovery after Adult Mammalian CNS Injury

**DOI:** 10.1523/ENEURO.0270-16.2016

**Published:** 2016-12-26

**Authors:** Xingxing Wang, Yuichi Sekine, Alexandra B. Byrne, William B.J. Cafferty, Marc Hammarlund, Stephen M. Strittmatter

**Affiliations:** 1 Program in Cellular Neuroscience, Neurodegeneration & Repair, Yale University School of Medicine, New Haven, CT 06536; 2 Department of Neuroscience, Yale University School of Medicine, New Haven, CT 06520; 3 Department of Neurology, Yale University School of Medicine, New Haven, CT 06520; 4 Department of Genetics, Yale University School of Medicine, New Haven CT 06520

**Keywords:** axon regeneration, optic nerve regeneration, PARP, poly (ADP-ribose), spinal cord injury

## Abstract

After traumatic damage of the brain or spinal cord, many surviving neurons are disconnected, and recovery of function is limited by poor axon regeneration. Recent data have suggested that poly ADP-ribosylation plays a role in limiting axonal regrowth such that inhibition of poly (ADP-ribose) polymerase (PARP) may have therapeutic efficacy for neurological recovery after trauma. Here, we tested systemic administration of the PARP inhibitor, veliparib, and showed effective suppression of PARylation in the mouse CNS. After optic nerve crush injury or dorsal hemisection of the thoracic spinal cord in mice, treatment with veliparib at doses with pharmacodynamic action had no benefit for axonal regeneration or functional recovery. We considered whether PARP gene family specificity might play a role. *In vitro* mouse cerebral cortex axon regeneration experiments revealed that short hairpin RNA (shRNA)-mediated suppression of PARP1 promoted axonal regeneration, whereas suppression of other PARP isoforms either had no effect or decreased regeneration. Therefore, we examined recovery from neurological trauma in mice lacking PARP1. No increase of axonal regeneration was observed in *Parp1*^–/–^ mice after optic nerve crush injury or dorsal hemisection of the thoracic spinal cord, and there was no improvement in motor function recovery. Thus, comprehensive *in vivo* analysis reveals no indication that clinical PARP inhibitors will on their own provide benefit for recovery from CNS trauma.

## Significance Statement

Poly (ADP-ribose) polymerase (PARP) inhibition has been proposed as a potential means to promote axonal regeneration and functional recovery after spinal cord injury or other CNS traumatic events. Here, pharmacologic and genetic methods were used to assess the potential of PARP as a therapeutic target, but no evidence for anatomical or behavioral benefit was observed after inhibition of PARP on its own.

## Introduction

After traumatic spinal cord injury, profound neurological deficits typically develop and persist chronically at all levels below the injury, despite the confinement of neuron loss to a much smaller zone of at-level damage. Loss of function derives substantially from the disconnection of surviving cells above and below the injury (Liu et al., 2006, 2011; Yiu and He, 2006; Schwab and Strittmatter, 2014). Thus, regeneration of severed but surviving axons has the potential to restore function. Unfortunately, little to no regeneration occurs in the adult mammalian CNS, including the spinal cord. In part, extracellular factors from oligodendrocyte, astroglial, and fibroblastic sources inhibit neuronal growth (Liu et al., 2006, 2011; Yiu and He, 2006; Schwab and Strittmatter, 2014). For example, overcoming myelin-derived inhibitors with a soluble Nogo receptor decoy therapeutic promotes axonal sprouting, regeneration, and neurological recovery even many months after trauma (Wang et al., 2006, 2011, 2014). In addition, the neurons of the adult CNS have limited cell autonomous propensity for growth (Liu et al., 2011; He and Jin, 2016). Increases of trophic factor signaling or elimination of endogenous brakes, such as PTEN phosphatase (Park et al., 2008) or other lipid phosphatases (Zou et al., 2015), can yield axonal regeneration in certain circumstances. Combinations of different effective methods provide limited overall recovery (Wang et al., 2012; Geoffroy et al., 2015, 2016; Jin et al., 2015), so additional pathways for targeted therapy are needed.

Two recent findings suggest that pharmacologic inhibition of poly (ADP-ribose) polymerase (PARP) *in vivo* might enhance neurological recovery. One study found that PARP activity was required for inhibitory factors such as Nogo and MAG to limit axon outgrowth in cultured neurons (Brochier et al., 2015). Further, PARP itself was found to be upregulated by CNS injury, suggesting it might function in axon regeneration (Brochier et al., 2015). A second study found that deletion or inhibition of PARPs did in fact enhance axon regeneration, both *in vivo* for the nematode *Caenorhabditis elegans* and *in vitro* for mammalian cerebral cortical cultures (Byrne et al., 2016). That study also found that the balance between PARPs and their counteracting enzymes, poly (ADP-ribose) glycohydrolase, is regulated by the conserved axon regeneration factor DLK (Byrne et al., 2016). Thus, multiple lines of evidence point to PARP as a potential *in vivo* target for improving mammalian CNS regeneration.

Assuming that PARP inhibition can support axonal regeneration, the pre-existence of pharmacologic tools to inhibit this enzyme class may provide a rapid transition to clinical testing and deployment. Here, we sought to test this potential. We used an orally available PARP inhibitor, veliparib, which targets several PARPs, including PARP1 (Wahlberg et al., 2012). It is being tested in phase 3 trials for breast, lung, and ovarian cancers. We observed inhibition of PAR levels in the retina by drug but not improvement in axon regeneration or recovery from optic nerve or spinal cord injury. Further investigation showed that among the PARP gene family, PARP1 suppression yielded the greatest regeneration in vitro. Therefore, genetic deletion of PARP1 gene was examined in the same injury models, but again, no benefit was observed. These data fail to reveal preclinical evidence for the use of PARP inhibitors in recovery from CNS trauma.

## Materials and Methods

### Animals

C57BL/6 mice (10–12 weeks of age, Jackson Laboratory cat. #JAX:000664 RRID:IMSR_JAX:000664), 129S-*Parp1^tm1Zqw^*/J (10–12 weeks of age, Jackson Laboratory cat. #JAX:002779 RRID:IMSR_JAX:002779), and 129S1/SvImJ mice (10–12 weeks of age, Jackson Laboratory cat. #JAX:002448 RRID:IMSR_JAX:002448) were used in this study (Wang et al., 1995). All experimental procedures were performed in compliance with animal protocols approved by the Institutional Animal Care and Use Committee at Yale University.

### Primary cortical neuron culture

Cortices from E17 C57BL/6 mice brain were dissected in ice-cold Hibernate E medium (cat. #HE-Ca; BrainBits) and incubated in digestion HBSS containing 30 U/ml Papain (cat. #LS003127; Worthington Biochemical), 1.5 mM CaCl2, 2.5 mm EDTA, and 2 mg/ml DNaseI (cat. #DN25; Sigma-Aldrich) at 37°C for 30 min. Digested tissues were triturated and suspended in Neurobasal-A medium supplemented with B-27, GlutaMAX, sodium pyruvate, and penicillin-streptomycin (all from Invitrogen). Cells were plated on 96-well tissue culture plates coated with poly-d-lysine at a density of 2.5 × 10^4^ cells per well in 200 μl of plating medium.

### Cortical axon regeneration assay (cortical scrape assay)

A cortical neuron scrape regeneration assay has been described in detail (Huebner et al., 2011; Zou et al., 2015). The use of lentiviral TRC1 shRNA library clones (The RNAi Consortium of the Broad Institute provided via Sigma-Aldrich) to suppress gene expression in these cultures has been documented (Zou et al., 2015). On day in vitro 3 (DIV3), 1 × 10^5^ TU of lentiviral particles targeting mammalian nontargeting (NC) short hairpin RNA (shRNA) control (cat. #SHC002V), *Parp1* shRNA (NMID: NM_007415, clone ID: TRCN0000071208, TRCN0000071209, TRCN0000071210, TRCN0000071211, TRCN0000071212; Sigma-Aldrich), *Parp2* shRNA (NMID: NM_009632, clone ID: TRCN0000071213, TRCN0000071214, TRCN0000071215, TRCN0000071216, TRCN0000071217; Sigma-Aldrich), *Parp3* shRNA (NMID: NM_145619, clone ID: TRCN0000093894, TRCN0000093895, TRCN0000093896, TRCN0000093897, TRCN0000093898; Sigma-Aldrich), *Parp9* shRNA (NMID: NM_030253, clone ID: TRCN0000174399, TRCN0000174697, TRCN0000173214, TRCN0000176202, TRCN0000175373; Sigma-Aldrich), *Parp12* shRNA (NMID: NM_172893, clone ID: TRCN0000174741, TRCN0000174854, TRCN0000175447, TRCN0000175542, TRCN0000175901; Sigma-Aldrich), or *Parp16* shRNA (NMID: NM_177460, clone ID: TRCN0000200923, TRCN0000190330, TRCN0000201597, TRCN0000190801; Sigma-Aldrich) were added to primary cortical neurons. On DIV8, 96-well cultures were scraped using a floating pin tool with FP1-WP pins (V&P Scientific) and allowed to regenerate for another 72 h before fixing with 4% paraformaldehyde PFA). Regenerating axons in the scrape zone were visualized using an antibody against βIII tubulin (1:2000, mouse monoclonal; cat. #G712A; Promega). Growth cones were visualized by staining for F-actin using rhodamine-conjugated phalloidin (1:2000, cat. #R415; Invitrogen). Cell density was visualized using nuclear marker 4′,6-diamidino-2-phenylindole (DAPI; 0.1 μg/mL, cat. #4083; Cell Signaling Technology). Images were taken on a 10× objective in an automated high-throughput imager (ImageXpress Micro XLS, Molecular Devices) under identical conditions. Regeneration zone identification, image thresholding, and quantitation were performed using an automated Matlab script.

### Veliparib treatment and immunoblotting

For the veliparib treatment study, C57BL/6 mice with or without optic nerve crush injury were treated once daily i.p. with veliparib (10 mg/kg/d, cat. #A3002; ApexBio Technology) or the same volume of normal saline as vehicle for 5 d beginning on the day of injury. Retina were dissected and sonicated in radioimmunoprecipitation assay (RIPA) buffer and centrifuged at 20,000 × *g* for 30 min. The pellet and lysate were resolved by SDS-PAGE, transferred to nitrocellulose membranes, immunoblotted with anti-poly (ADP-ribose) (1:1000, cat. #4335-MC-100, RRID: AB_2572318, Trevigen), anti–β-actin (1:3000, cat. #8457, RRID: AB_10950489, Cell Signaling Technology), and anti–β-tubulin (1:2000, cat. #sc-55529, AB_2210962, Santa Cruz Biotechnology) primary antibodies. After primary antibody incubation, secondary antibodies (Odyssey IRDye 680 or 800) were applied for 1 h at room temperature. Membranes were then washed and visualized using a Licor Odyssey Infrared imaging system. For *Parp1* mutant mice study, 129S-Parp1^tm1Zqw/J^ and 129S1/SvImJ mice retinas were analyzed by the same method as described above.

### Reverse transcription PCR and quantitative PCR

Total RNA from mouse cortex, spinal cord, and retina were prepared according to the TRIzol Reagent protocol (Sigma-Aldrich) and subjected to reverse transcriptase (RT)-PCR using M-MuLV Reverse Transcriptase (New England Biolabs). Complementary DNA (cDNA) of each sample was then used for real-time quantitative PCR with iQ supermix (Bio-Rad) and TaqMan Gene Expression Assay (#Mm01321084 for *Parp1*, #Mm00518778 for *Parp9*, and #Mm03302249 for *Gapdh* from Applied Biosystems) on a Bio-Rad CFX Connect Real-Time PCR Detection System using standard cycles. Each sample was loaded in triplicate.

### Optic nerve crush injury

For the veliparib treatment study, male C57BL/6J mice were anesthetized by i.p. injection of ketamine (100 mg/kg) and xylazine (10 mg/kg). Topical 2% lidocaine anesthetic was applied to the eyeball. The optic nerve (ON) was exposed intraorbitally with care taken to avoid damage to the ophthalmic artery. The nerve was injured by crushing with a jeweler’s forceps (Dumont 5; Fine Science Tools) for 10 s at a location 1 mm posterior to the eyeball (Wang et al., 2011, 2015). Three days after injury, mice were treated once daily i.p. with veliparib (10 mg/kg/d) or the same volume of normal saline as vehicle. After 14 d of treatment, Alexa-555-Cholera toxin beta (CTB) was injected intravitreally to trace retinal ganglion cell axons. Three days after CTB, mice were killed by transcardial perfusion with PBS followed by 4% PFA. Previous published experiments used identical methods and detected regenerating axons with various manipulations including zymosan (Wang et al., 2011b; Duffy et al., 2012; Wang et al., 2012; Wang et al., 2015).

For ON regeneration study in *Parp1* mutant mice, cohorts of 129S-*Parp1^tm1Zqw^*/J and 129S1/SvImJ mice were anesthetized, and ON was crushed by the same method as described above. Alexa-CTB was injected intravitreally 14 d after injury, and tissues were collected 3 days later.

### Thoracic dorsal hemisection injury

Female mice were first anesthetized with 4% isoflurane and maintained with 2% isoflurane throughout the procedure. A laminectomy was performed to expose the dorsal portion of spinal cord corresponding to T8 and T9 levels. The spinal dorsal hemisection was performed at T8 level with a pair of microscissors to a depth of 1.1 mm to completely sever the dorsal and dorsolateral corticospinal tract (CST). Lateral aspect of the spinal cord was scraped with a 30-gauge needle to ensure completeness of the lesion. Muscle and skin overlying the lesion were sutured with 4-0 vicryl. All animals received subcutaneous injection of 100 mg/kg ampicillin and 0.1 mg/kg buprenorphine twice a day for the first 2 d after surgery. For the veliparib treatment study, animals were treated once daily i.p. with veliparib (10 mg/kg/d) or the same volume of normal saline as vehicle beginning at day 3 postinjury and continuing for 28 d. To trace the CST, mice were reanesthetized with i.p. injection of ketamine and xylazine 6 weeks after spinal cord injury (SCI). Biotin dextran amine (BDA; 0.1 g/ml in sterile normal saline, Thermo Fisher Scientific cat. # D1956 RRID:AB_2307337) was injected into the sensorimotor cortex to anterogradely label the CST (Fink et al., 2015). Two weeks after the tracing, animals were killed by transcardial perfusion with PBS followed by 4% PFA.

### Behavioral testing

For mouse behavioral observation, the Basso mouse scale (BMS) was used (Basso et al., 2006). All behavioral tests were performed by two researchers unaware of the genotype of the mice or the identity of the compound in the syringe. Observations were made once per week.

### Histology and immunohistochemistry

#### Optic nerve

The ON was dissected from the eyeball and postfixed in the 4% PFA solution. After treating the whole nerve with an optical clearing procedure (Erturk et al., 2011), the sample was mounted on a glass side with a coverslip for imaging.

#### Spinal cord

The spinal cord was dissected and postfixed in the 4% PFA solution. The spinal cord segment 5 mm rostral to 5 mm caudal of the lesion center was embedded in 10% gelatin and postfixed in the 4% PFA solution at 4°C for 48 h. Serial parasagittal sections were collected at a thickness of 40 µm on a vibrating microtome. BDA detection was performed using a tagged avidin reaction (Vectastain ABC; Vector Laboratories cat. # PK-7100 RRID:AB_2336827) and following protocols of the TSA cyanine 3 amplification system (PerkinElmer, cat. # NEL704A001KT RRID: AB_2572409).

For ON axon quantification, the entire ON was imaged with a Zeiss LSM 710 confocal microscope with a 20× objective using a *z*-stack of 4-µm steps and an *XY* montage. Axons labeled with CTB crossing transverse lines at specific distances of 100, 200, 500, and 1000 µm central to the crush site were counted by an observer unaware of experimental group throughout the entire *z*-stack at each specified distance along the optic nerve.

For BDA-labeled CST axon quantification in spinal cord, every other section from serial sagittal sections from each spinal cord sample were imaged using a 10× objective on a Z1 Imager microscope (Zeiss). For separate dorsal-ventral lines at 3, 2.5, 2, 1.5, 1, and 0.5 mm rostral to the lesion center and 0.5, 1, 1.5, and 2 mm caudal to the lesion in each section, the number of BDA-positive fibers crossing the line was counted by an observer unaware of experimental group. The sum of all axons per animal was multiplied by a factor of 2, because every second section was analyzed. Data represent the number of BDA-labeled axons per mouse ± SE.

### Statistics

All data were analyzed with SPSS (SPSS Inc.) or Microsoft Excel (Microsoft Corp.) software. Superscript letters listed with *p*-values correspond to the statistical tests shown in Table 1.

**Table 1.** Statistical analyses used in this study.

Line	Data structure	Type of test	*P*
a	Normal distribution	One-way ANOVA, with Tukey post hoc pairwise tests	0.0004
b	Normal distribution	One-way ANOVA, with Tukey post hoc pairwise tests	0.0032
c	Normal distribution	One-way ANOVA, with Tukey post hoc pairwise tests	0.04
d	Normal distribution	One-way ANOVA, with Tukey post hoc pairwise tests	0.0001
e	Normal distribution	One-way ANOVA, with Tukey post hoc pairwise tests	0.0014
f	Normal distribution	One-way ANOVA, with Tukey post hoc pairwise tests	0.0001
g	Normal distribution	One-way ANOVA, with Tukey post hoc pairwise tests	0.0001
h	Normal distribution	One-way ANOVA, with Tukey post hoc pairwise tests	0.0001
i	Normal distribution	One-way ANOVA, with Dunnett post hoc pairwise tests	0.0001
j	Normal distribution	One-way ANOVA, with Dunnett post hoc pairwise tests	0.0017
k	Normal distribution	One-way ANOVA, with Tukey post hoc pairwise tests	0.0001
l	Normal distribution	One-way ANOVA, with Tukey post hoc pairwise tests	0.0001
m	Normal distribution	One-way ANOVA, with Tukey post hoc pairwise tests	0.0001
n	Normal distribution	Student’s two tailed *t* test	0.046

## Results

### Pharmacologic PARP inhibition

**Figure 1.**
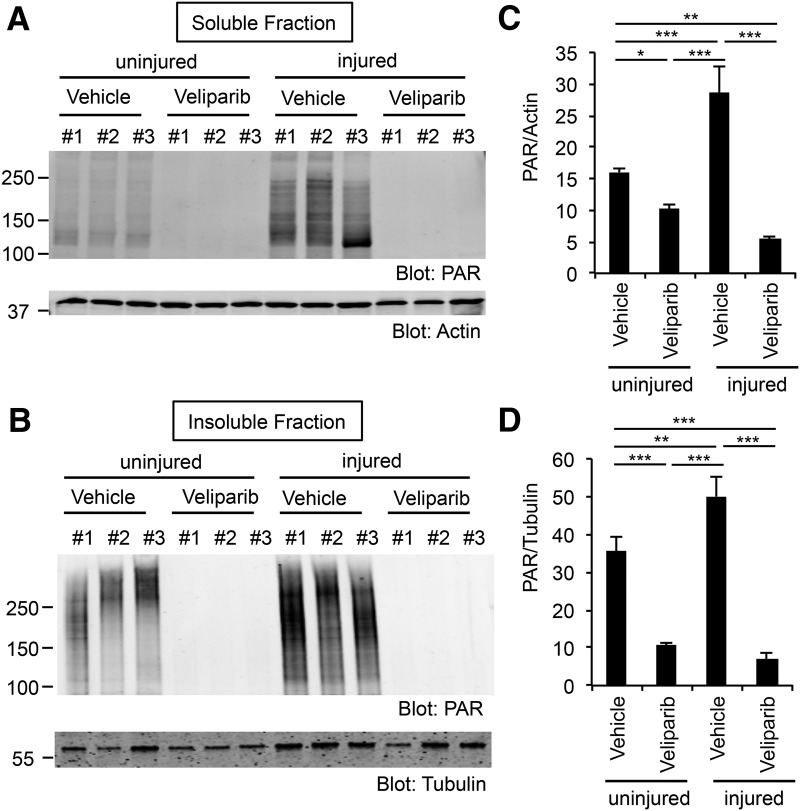
PARylation is induced after ON injury and suppressed by veliparib treatment in retina tissue. ***A***, ***B***, Immunoblots show PARylation in retinal tissue from naive state or 5 d after ON crush and treated with veliparib (10 mg/kg/d) or vehicle. RIPA soluble (***A***) and insoluble (***B***) samples were analyzed with an antibody directed against PAR protein. Molecular weight markers in kDa are at left. ***C***, ***D***, Quantification of PARylation in the lysate (***C***) or pellet (***D***). ON crush injury significantly increased PARylation in retina, and veliparib treatment significantly suppressed it. Data are mean ± SE from *n* = 3 in each condition. For one-way ANOVA followed by post hoc pairwise Tukey’s tests: *, *P* <0.05; **, *P* <0.01; ***, *P* <0.005.

To explore the potential of PARP as a therapeutic target, we treated mice with the inhibitor veliparib (ABT-888), a PARP inhibitor with high potency for at least PARP1, PARP2, PARP3, and PARP4 (Wahlberg et al., 2012). Using 10 mg/kg/d daily i.p. doses, we evaluated the success of CNS target engagement by monitoring the level of protein poly ADP-ribosylation (PARylation) from immunoblots of retinal tissue with an anti-PAR antibody. RIPA soluble and insoluble fractions from retina of naive mice or mice with ON crush 5 d previously were analyzed. PARylated protein was readily detected in lysates of control mice as multiple molecular weight species >120 kDa in soluble and insoluble fractions (Fig. 1*A*, *B*). There was a significant increase in PAR levels by 50–100% in both biochemical fractions in the retina from ON crush mice relative to naive mice (Fig. 1*A–*D, *P* = 0.0004^a^ soluble and *P* = 0.0032^b^ insoluble). This PARylation increase is similar to that described in Brochier et al. (2015). After 5 d of veliparib administration to naive mice, the level of PARylated protein was suppressed within the retinal tissue (Fig. 1*A–D*, *P* = 0.04^c^ soluble and *P* = 0.0001^d^ insoluble). The reduction was even more pronounced in ON crush mice treated with veliparib relative to naive vehicle group or ON crush vehicle group (Fig. 1*A–D*, *P* = 0.0014^e^ soluble and *P* = 0.0001^f^ insoluble relative to naive vehicle; *P* = 0.0001^g^ soluble and *P* = 0.0001^h^ insoluble relative to ON crush vehicle). For ON crush injured retina, veliparib reduced PAR levels by >80%.

**Figure 2.**
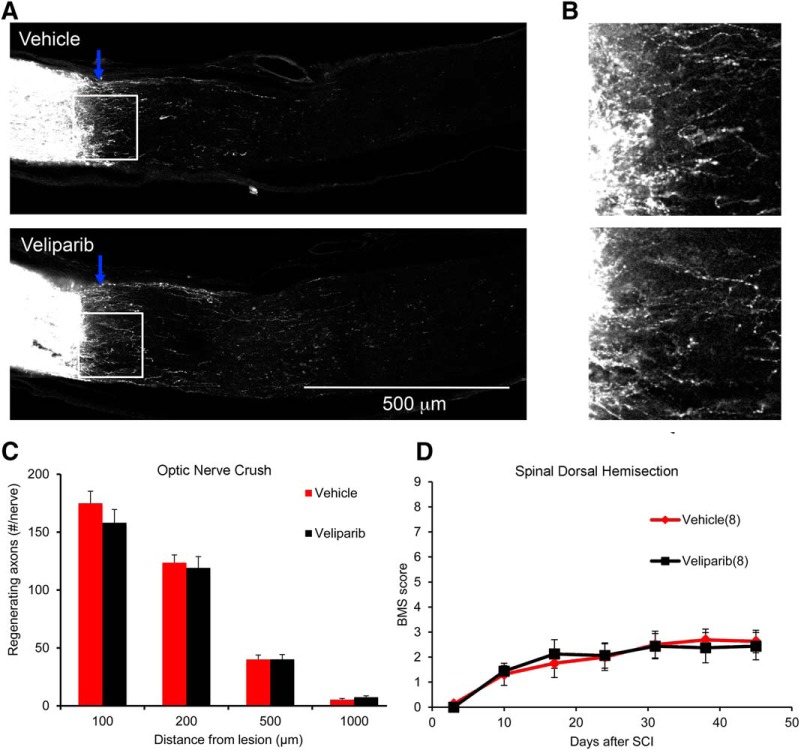
Pharmacologic PARP inhibition does not increase ON axon regeneration or improve functional recovery from SCI. ***A***, Mice underwent ON crush injury and were treated with veliparib or vehicle on d 3–17 postinjury. Representative images of ON from vehicle-treated and veliparib-treated mice. Multiple CTB-labeled axons proximal to the crush site are observed. Images are projections of confocal *z*-stacks through the entire ON. The eye is to the left, and the crush site is indicated by the blue arrow. Few axons extend centrally. ***B***, High-magnification view of the lesion area (box) from ***A***. ***C***, Total number of regenerating ON fibers per mouse is presented as a function of distance central to the crush site. There is no statistically significant difference in the number of regenerating axons between the saline-treated group and the veliparib-treated group. Data are mean ± SE of *n* = 8 mice per group. ***D***, Mice underwent midthoracic dorsal hemisection and then were treated with veliparib or vehicle on d 3–31 postinjury. The locomotor BMS score is plotted as a function of time after SCI. There is no statistically significant difference in the number of regenerating axons between the saline-treated group and the veliparib-treated group by one-way repeated-measure ANOVA. Data are mean ± SE for *n* = 8 mice per group.

ON regeneration after retroorbital crush injury was assessed in mice with PARP inhibition (Fig. 2). The compound was administered beginning 3 d postinjury, to mimic a clinically relevant time frame, and doses were continued once daily for 14 d. The retinal ganglion cell (RGC) axons were anterogradely labeled by intraocular injection of CTB on d 17 postinjury, and tissue was collected 3 d later (d 20) for analysis of axon regeneration. Labeling of the ON between the eye and the crush was robust (Fig. 2*A*, *B*), but few fibers extended past the crush site (Fig. 2*A*, *B*), and there was no difference in the axon counts between groups (Fig. 2*C*). Thus, PARP inhibition fails to promote RGC axonal regeneration.

ON regeneration provides anatomical assessment of CNS axon regeneration, but spinal cord trauma with motor function assessment has greater clinical translation impact. Thus, we created midthoracic dorsal hemisection injuries and treated mice with veliparib beginning 3 d after SCI and continuing for 28 d, using the same dosing strategy as for the ON studies. Functional outcome was surveyed by the BMS score to monitor locomotion in the open field (Fig. 2*D*). Recovery of hindlimb function was indistinguishable between the veliparib and vehicle control groups. Axonal anatomy was not assessed in this cohort, which lacked evidence for functional improvement. The findings provide further evidence against PARP inhibition providing a target for CNS trauma therapy.

### PARP specificity in axonal regeneration

**Figure 3.**
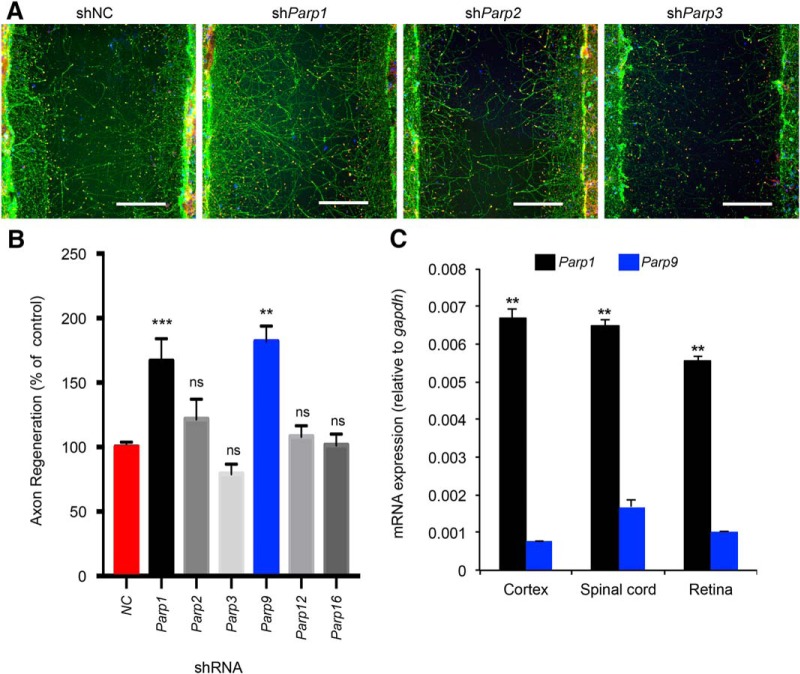
Selective suppression of *Parp1* improves cortical neuron axonal regeneration. ***A***, Representative images of regenerated cortical neurons. Neurons were transduced with shRNA lentiviral particles on culture d 3 and scraped on d 8. Scraped neurons were fixed and stained at d 11. The microphotographs illustrate βIII tubulin in axons (green), phalloidin of F-actin in growth cones (phalloidin, red), and cell nuclei (DAPI, blue). Note that no neuronal cell bodies (DAPI) migrate into the scrape zone. Selectively after *Parp1* knockdown, a greater number of regenerating axons and growth cones are visible in the scrape zone. Scale bars, 200 µm. ***B***, Quantification of axon regeneration. Data are mean ± SE. shNC, *n* = 109; sh*Parp1*, *n* = 49 (each of 5 lentiviral species has 10 independent wells, with 1 cell culture lost); sh*Parp2*, *n* = 10 (each of 5 lentiviral species has 2 independent wells); sh*Parp3*, *n* = 10 (each of 5 lentiviral species has 2 independent wells); sh*Parp9*, *n* = 10 (each of 5 lentiviral species has 2 independent wells); sh*Parp12*, *n* = 10 (each of 5 lentiviral species has 2 independent wells); and sh*Parp16*; *n* = 16 (each of 4 lentiviral species has 4 independent wells). Each condition was compared to NC control by one-way ANOVA followed by Dunnett test. **, *P* <0.01; ***, *P* <0.005; ns, no significant difference. ***C***, Quantification of *Parp1* and *Parp9* mRNA. The levels of the *Parp1* and *Parp9* mRNA determined by quantitative RT-PCR were normalized to those of a GAPDH internal control for cerebral cortex (*n* = 3 mice), spinal cord (*n* = 3), and retina (*n* = 3). Data are mean ± SE. Within each tissue, *Parp1* was significantly greater than *Parp9* for each tissue after one-way ANOVA with post hoc pairwise Tukey test. **, *P* <0.0001.

The mammalian PARP family is large, with 17 members (Amé et al., 2004; Wahlberg et al., 2012). We considered the possibility that inhibition of certain PARP enzymes might promote axon regeneration whereas others might have no effect or opposite action. To explore PARP specificity, we cultured cerebral cortical neurons, allowed a lawn of axons to extend, and mechanically axotomized a region in the center of a microtiter well. Under control conditions, a limited percentage of axons regenerate across the scraped gap, whereas few if any cells migrate into the injured area (Huebner et al., 2011; Zou et al., 2015). Axons can be visualized by anti–βIII-tubulin staining, and their growth cones, by phalloidin staining for filamentous actin (Fig. 3*A*). We used lentiviral preparations encoding shRNAs targeting specific PARPs driven by the U6 promoter to knock down different PARP enzyme mRNAs (Fig. 3*A*). For each PARP, four to five different shRNAs targeting sequences were applied in different wells, and the results were averaged for each gene (Fig. 3*B*). *Parp1* knockdown yielded a significant (Fig. 3*B*, *P* = 0.0001^i^) two-fold increase in axonal regeneration relative to control, whereas most other PARP species produced little change in regeneration (Fig. 3*A*, *B*). *Parp9* suppression also increased regeneration relative to nontargeting control shRNA (Fig. 3*B*, *P* = 0.0017^j^).

We considered the expression pattern of *Parp1* and *Parp9* in the adult CNS as an indication as to whether one or both might be relevant for repair. The mRNA levels measured by quantitative RT-PCR in brain, spinal cord, and retina are 5–10 times higher for *Parp1* than for *Parp9* (Fig. 3*C*, *P* = 0.0001 for cortex,^k^ spinal cord,^l^ and retina^m^). We concluded that, among the gene family, *Parp1* is most likely to be relevant for adult CNS axonal regeneration.

### Axon regeneration in *Parp1*-null mice

**Figure 4.**
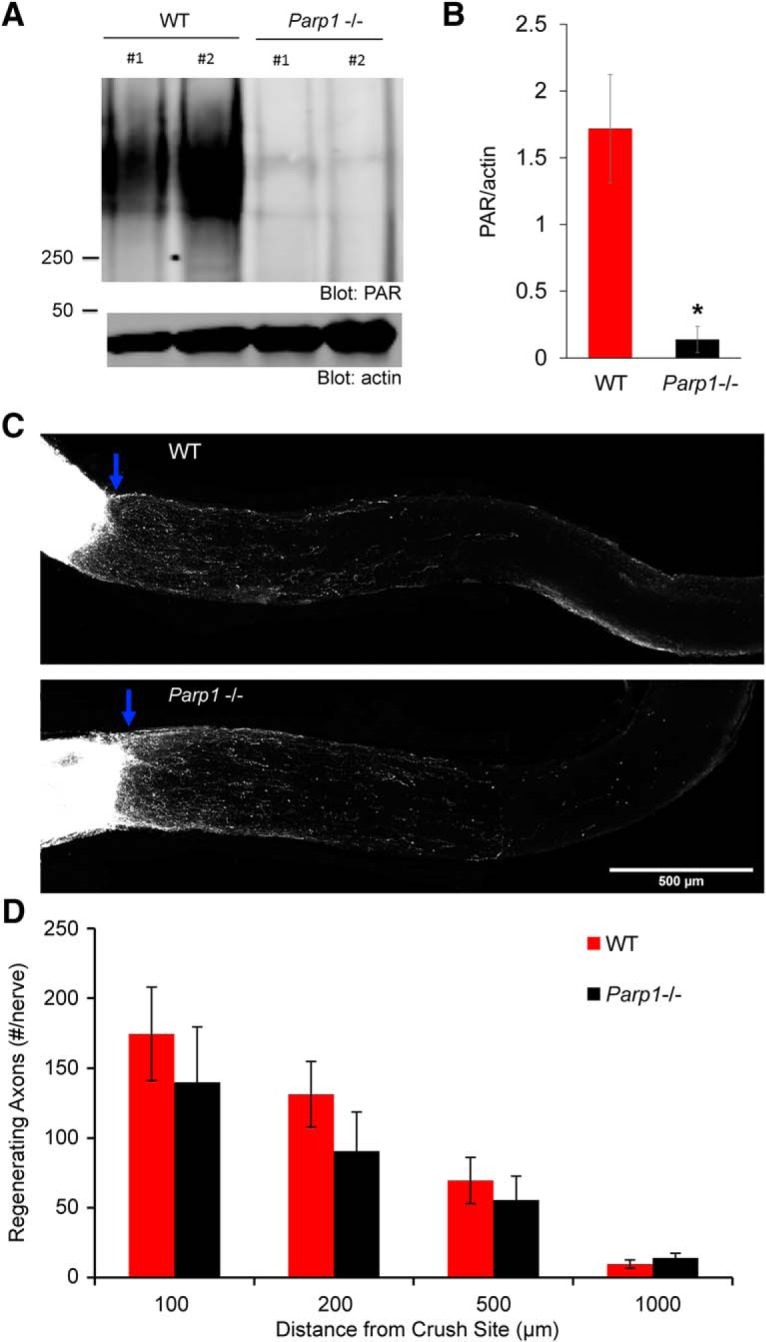
Genetic deletion of *Parp1* does not increase ON axon regeneration. ***A***, Immunoblots are shown for PARylation levels from uninjured wild-type (WT) control mice or *Parp1*^–/–^ mutant mice in RIPA-insoluble fraction of retinal tissue. Molecular weight markers in kDa are at left. ***B***, Quantification of PARylation in retinal tissue from WT mice and *Parp1*-null mutant mice from blots as in ***A***. Data are mean ± SE for *n* = 2 mice per genotype. *, *P* = 0.046, Student’s two-tailed *t* test. ***C***, Representative images of ON from WT control mouse and *Parp1* mutant mouse. The CTB-labeled RGC axons are white. The eye is the left and the brain to the right, with crush indicated by blue arrow. Images are projections of confocal *z*-stacks through the entire ON. Scale bar, 500 µm. ***D***, The total number of regenerating optic nerve fibers per mouse is presented as a function of distance central from the crush site and of genotype. Data are mean ± SE for *n* = 8 WT and *n* = 8 *Parp1*-null mice. No statistically significant difference was observed between the *Parp1*^–/–^ and WT mice by one-way repeated-measure ANOVA.

Based on the shRNA and expression results, we chose to examine the role of *Parp1* in axonal regeneration by genetic methods. An existing line of mice null for *Parp1* expression (Wang et al., 1995) was studied in CNS trauma models. First, we assessed PARylation in immunoblots of retinal tissue, with comparison to the effects of veliparib treatment. Remarkably, deletion of *Parp1* alone suppressed the vast majority of PARylation in the retinal tissue (*P* = 0.046^n^, Fig. 4*A*, *B*). We subjected *Parp1*^–/–^ and strain- and background-matched WT mice to ON crush injury, with regeneration analysis as for veliparib studies by CTB tracing. Intense axonal labeling was observed to fill axons up to the crush site, but few fibers extended past the injury in either genotype (Fig. 4*C*). There was no detectable difference in axonal counts central to the injury site (Fig. 4*D*), indicating that endogenous *Parp1* does not limit RGC axonal regeneration.

**Figure 5.**
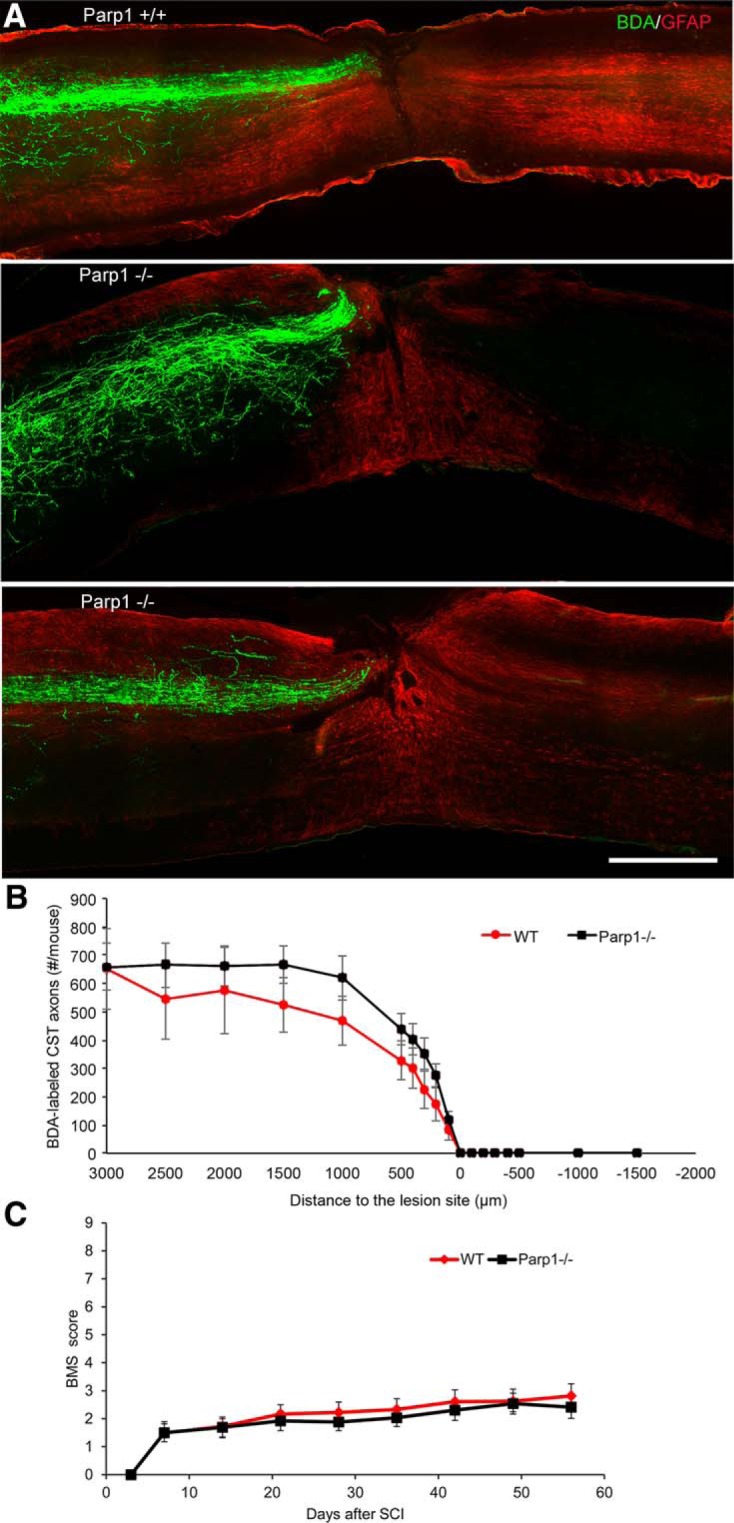
Genetic deletion of *Parp1* does not improve recovery from dorsal hemisection of thoracic spinal cord in mice. ***A***, Wild-type (WT) and Parp1^–/–^ mice underwent midthoracic dorsal hemisection injury. Representative images of a spinal cord sagittal section from one WT mouse and two *Parp1*-null mutant mice collected 6 weeks after injury. CST axons are visualized by BDA tracing (green) and astrocytic reaction by anti-GFAP staining (red). The entire depth of the spinal cord is shown. Rostral is to the left, and dorsal is up. No BDA-labeled axons are seen caudal to the lesion site in either group. Scale bar, 500 µm. ***B***, Quantification of BDA-labeled CST axons rostral and caudal to the lesion site. For the *x*-axis, a positive value is rostral to the center of the lesion, and a negative value is caudal to the center of the lesion. Data are mean total number of CST axons per mouse at each location ± SE for *n* = 8 WT mice and *n* = 12 *Parp1*-null mice. No statistically significant difference was observed between the *Parp1*^–/–^ and WT mice by one-way repeated-measure ANOVA. ***C***, Open-field locomotion performance measured by BMS of WT and *Parp1*^–/–^ mutant mice. Data are mean ± SE for *n* = 9 WT mice and *n* = 13 *Parp1*-null mice. No statistically significant difference was observed between the *Parp1*^–/–^ and WT mice by one-way repeated-measure ANOVA.

The *in vitro* axon regeneration suggests a role for *Parp1* in cortical neurons, so we also studied recovery from SCI in *Parp1*-null mice. Adult mice underwent midthoracic dorsal hemisection injury, and the CST was traced anterogradely by cortical injection of BDA 6 weeks after SCI. After 2 weeks of tracing, mice were killed, and evidence for CST axon regeneration was assessed. Although many CST fibers were detected rostral to the injury, none regenerated past the injury site in either genotype (Fig. 5*A*). Counts of BDA-labeled CST axons showed indistinguishable degrees of axonal dieback and lack of axonal regeneration or sprouting in the two groups (Fig. 5*B*). The motor performance of this cohort was monitored by BMS scoring for a total of 7 weeks after injury. The recovery of *Parp1*^–/–^ mice was indistinguishable from that of control mice (Fig. 5*C*).

## Discussion

The major finding of this study is that PARP inhibition or deletion fails to support axon regeneration *in vivo*. Using genetic and pharmacologic interventions, we observed suppression of CNS PARylation, so the interventions appeared to achieve the desired biochemical action. However, despite previously published studies (Brochier et al., 2015; Byrne et al., 2016) and the *in vitro* data obtained here, we observed no axon regeneration effects *in vivo*. The lack of PARP inhibition and *Parp1* deletion was observed in two different models of CNS trauma, namely the ON crush and the spinal cord hemisection trauma. Thus, the current preclinical studies do not support pursuit of PARP inhibition as a means to promote neurological recovery from CNS trauma.

For *in vitro* systems and invertebrate systems, PARP inhibition effectively stimulates axonal regeneration. The failure of PARP inhibition or *Parp1* deletion after adult mammalian CNS injury implies factors specific to the more complex injury system. This may include the presence of glial inhibition by myelin-derived inhibitors, fibroblast scars, or astrocytic reaction to injury. Regardless, the findings emphasize the need to test results from model systems in the adult mammal to confirm translational relevance. It remains possible that PARP inhibition would function synergistically with other axonal growth-promoting strategies in a combinatorial approach, even though monotherapy is unlikely to have benefit.

A striking finding is that *Parp1* knockdown, but not that for several other PARP enzymes, promotes cortical regeneration *in vitro*. The basis of this specificity may stem in part from differential expression of the PARP enzymes themselves. Although *Parp9* knockdown suppressed in cortical neuron cultures, its expression is much lower than that of *Parp1* in adult CNS tissue, and *Parp1* deletion eliminates nearly all PARylation in retina. It remains possible that constitutive *Parp9/Parp1* double-gene deletion mice would exhibit substantial axonal regeneration even though effective veliparib inhibition did not do so beginning 3 d after injury. The data also suggest that neuronal poly (ADP-ribose) substrates of *Parp1* are more relevant for regeneration than sites modified by other PARPs. Proteomic analysis of substrate specificity may provide a means to identify regeneration-relevant pathways downstream of PARP activity.

Pharmacologic inhibition was initiated at 3 d postinjury in our experiments. It remains possible that earlier inhibition with a broad-spectrum PARP isoform inhibitor might promote axonal regeneration or generate neuronal protection. However, earlier intervention is translationally challenging and should not be required to achieve success with a reparative strategy. At this stage, we conclude that translational development of PARP inhibition is unlikely to be effective for SCI monotherapy.

## Synthesis

Reviewing Editor: Michael Sofroniew, UCLA School of Medicine Decisions are customarily a result of the Reviewing Editor and the peer reviewers coming together and discussing their recommendations until a consensus is reached. When revisions are invited, a fact-based synthesis statement explaining their decision and outlining what is needed to prepare a revision will be listed below. The following reviewer(s) agreed to reveal their identity: Jae Lee, Larry Benowitz.

Please add to the end of the significance statement "...was observed after inhibition of PARP on its own".

Please insert into the last sentence of the abstract (line 17) "...PARP inhibitors will on their own provide..."

The additions to the significance statements and last sentence of the abstract are made in response to a comment by one of the reviewers who points out that the authors have not ruled out that a combination of PARP inhibition with other approaches might have benefit. To leave open this possibility the additional wording was requested. All reviewers agreed.
